# In Response

**DOI:** 10.4269/ajtmh.2010.10-0130b

**Published:** 2010-08-05

Dear Sir:

We would like to thank the authors for their insights. We agree that serologic testing performed on acute serum or cerebrospinal fluid (CSF) specimens may not rule out a diagnosis of Japanese encephalitis (JE) and that we very likely underestimated disease burden. Although lower levels of JE virus (JEV) immunoglobulin (Ig) M antibodies in serum or CSF have been associated with poor outcome,^1^ most JE patients develop IgM antibodies in serum within 10 days after their onset of illness and in CSF by 7 days after their illness onset.^2^,^3^ JEV-specific IgM antibodies usually persist for at least several weeks.

In our study of 472 encephalitis patients with no evidence of recent JEV infection by antibody testing, 361 (77%) had negative JEV IgM-capture enzyme-linked immunosorbent assay (ELISA) testing on serum specimens collected ≥ 10 days after onset of illness. Of the remaining 111 JEV IgM-negative patients who only had a serum specimen obtained < 10 days after illness onset, 22 (20%) had negative JEV IgM ELISA testing on a CSF specimen that was collected between 7 and 20 days after the onset of their illness. Therefore, of the 492 patients evaluated during this study, 20 (4%) had laboratory evidence of recent JEV infection, and 383 (78%) had JE ruled out as the cause of their encephalitis. Although the remaining 89 (18%) patients had negative JEV IgM testing on serum and/or CSF specimens, JE could not be definitively ruled out as the cause of their illness (i.e., JEV unknown). We chose to conservatively assess burden by determining the proportion of individuals with positive laboratory findings over all individuals who had testing, regardless of the specimen timing. However, if the 89 patients who may be considered JEV unknown were as likely as those with optimally timed specimens to have JE (20/403; 5%), approximately five additional JE cases could have been added to the burden calculation.

This is the first study to document human JEV infection in Bangladesh since 1977. However, because of the methods used (i.e., every fourth patient meeting the clinical case definition for encephalitis was selected for laboratory evaluation) and limitations in case finding and laboratory testing, we likely captured a small proportion of JE cases occurring within the surveillance area during the study period, further contributing to a substantial underestimation of disease incidence. The findings of this study indicate that JEV infection is a potentially important cause of vaccine-preventable encephalitis in Bangladesh and warrants study to further characterize the epidemiology of JEV infection.

M. J. Hossain

E. S. Gurley

S. Montgomery

L. Petersen

J. Sejvar

M. Fischer

A. Panella

A. M. Powers

N. Nahar

A. K. Rafique Uddin

M. E. Rahman

A. R. Saifuddin Ekram

S. P. Luby

R. F. Breiman

E-mail: jhossain@icddrb.org
